# Supplementary material for: Model‐guided combinatorial optimization of complex synthetic gene networks
Source: Mol Syst Biol. 2016 Dec 28;12(12):899. doi: 10.15252/msb.20167265 (PMC5199127; doi:10.15252/msb.20167265)
Supplement: Supplementary file 1 — Expanded View Figures PDF [file MSB-12-899-s001.pdf]

Expanded View Figures

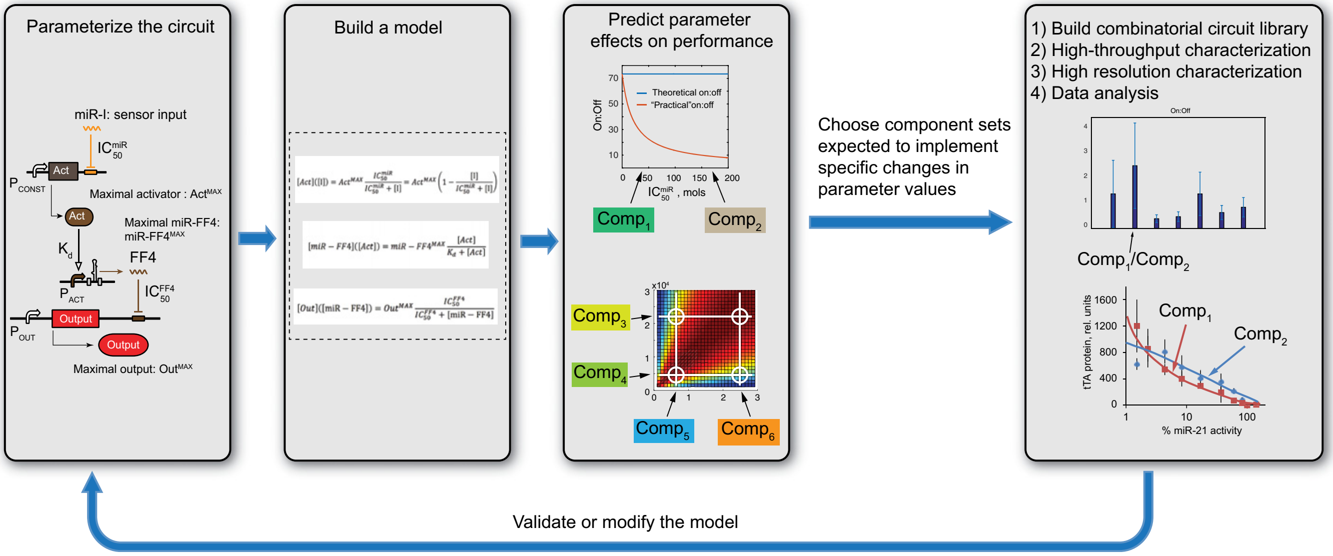

Figure EV1. Schematic representation of the integrated computational-experimental workflow.
From left to right: the circuit in question is parameterized; a model is built to describe relevant circuit outputs, in our case, the output level in the steady state; the model is analyzed to deliver predictions regarding optimal parameter regimes and the performance sensitivity to parameter changes; a circuit library is constructed with each functional block instantiated with at least two structural variants, implementing distinct parameter values; the library is evaluated experimentally in a high-throughput experiment and select circuits are studied in detailed low-throughput measurements to either validate or modify the model.

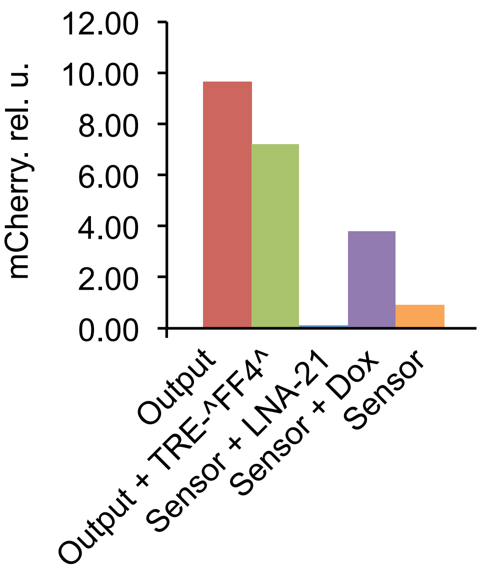

Figure EV2. Initial experimental characterization of a compact sensor.
The sensor comprises CMV-tTA-3′-T21, TRE-^miR-FF4^, and EF1A-mCherry-T302B-T136-TF. The bars, from left to right, are as follows: output (shows the expression of the output construct alone); output + TRE-^FF4^ (shows the expression of the output combined with the miR-FF4 cassette showing some leakage from the TRE promoter); sensor + LNA-21 (the Off state of the sensor obtained with LNA-21); sensor + Dox (the simulated on-state obtained with doxycycline and resulting in tTA inactivation); sensor (the On state triggered by endogenous miR-21 in HeLa cells). Although there is a strong LNA-21-mediated repression, the recovery in the On state is very poor. These measurements were performed once, but similar data were obtained in other preliminary experiments.

© 2016 The Authors

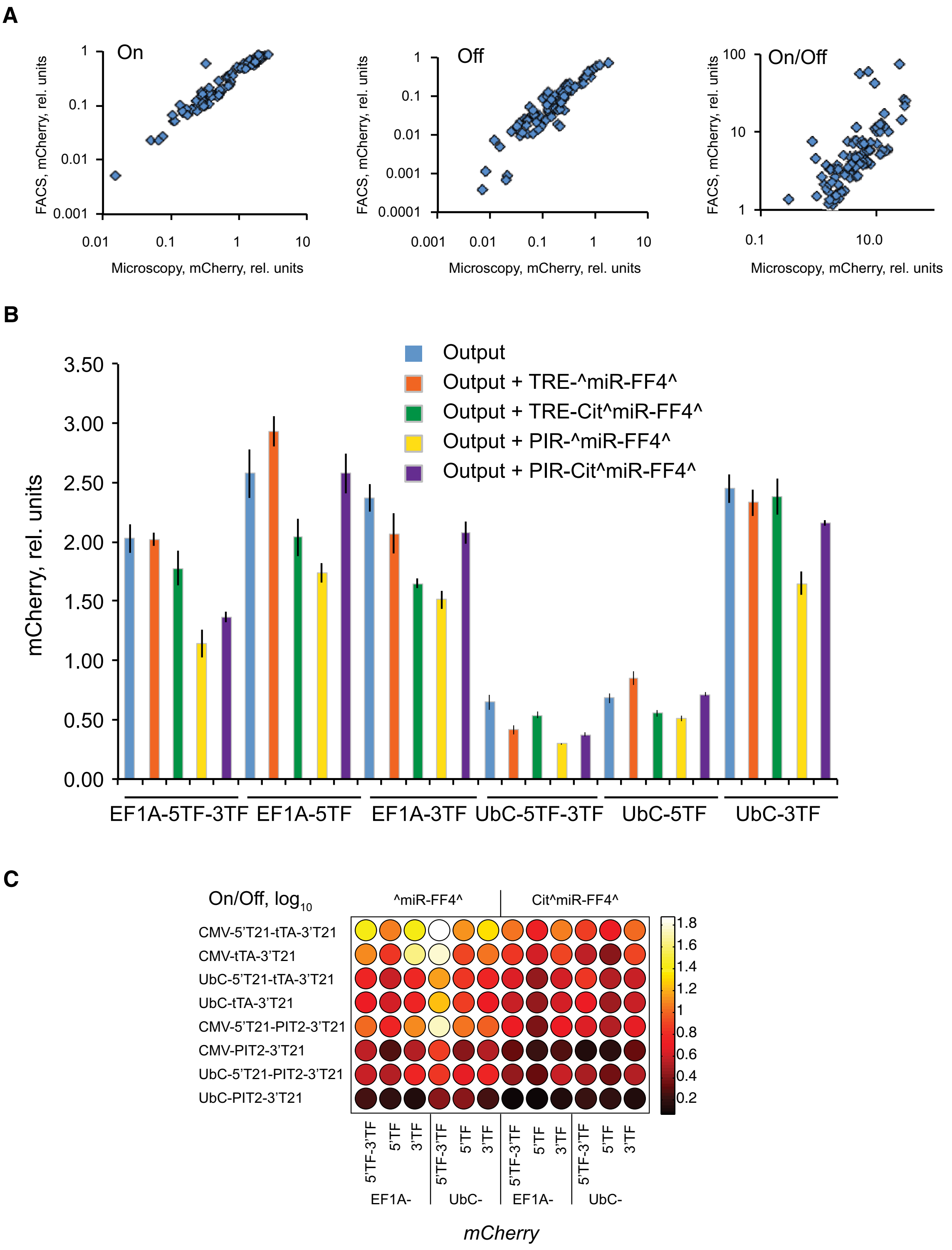

Figure EV3. Microscopy and flow cytometry analysis of screening results.

A Comparison of microscopy and flow cytometry results in On state, Off state, and On:Off ratio.

B Control measurements for all six output plasmids alone and together with upstream miRNA-expressing constructs as indicated by the color code in the legend.

C Heat maps depicting the flow cytometry analysis results in a 96-well plate format. Sensor activator and output components for each "well" are indicated.

Data information: Flow cytometry measurements (A, C) were performed once using one of the three replicate samples generated during the automated screening campaign. In (B) imaging-derived data represent a mean and a standard deviation of a triplicate measurement.

 © 2016 The Authors

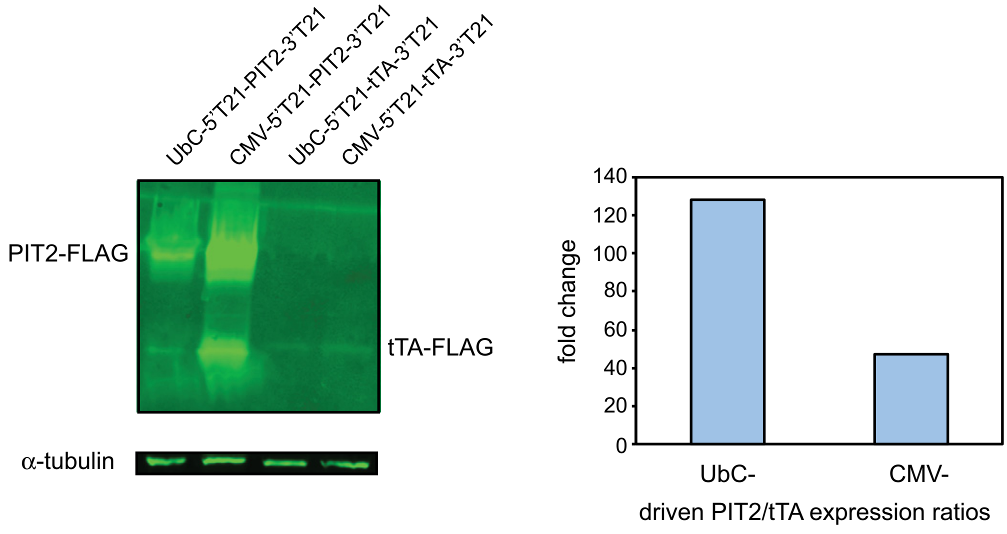

Figure EV4. Western blots of FLAG-tagged PIT2 and tTA constructs.
Bands in lanes, which were loaded with protein extracts from cells transfected with PIT2-FLAG constructs, migrating with a similar velocity as tTA-FLAG proteins in the other lanes are likely to represent degradation products of the full-length PIT2-FLAG fusion protein. In the right panel, a densitometric quantification of the α-tubulin normalized expression ratios between PIT2 and tTA is shown.

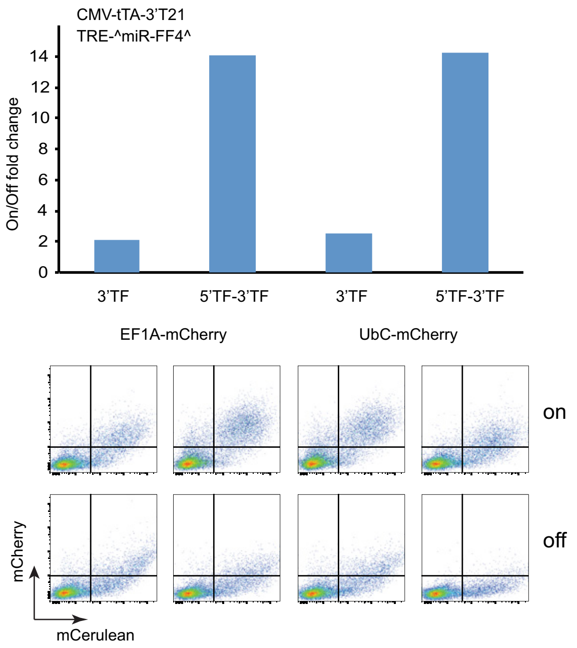

Figure EV5. Experimental comparison of 3′ and 5′+ 3′ output constructs.
HCT-116 were transfected with CMV-tTA-3′T21, a TRE-^miR-FF4^ and 3′- or 5′+3′-UTR mCherry output constructs using either EF1a or UbC promoters, as indicated. The fold-change of On:Off ratios are depicted together with exemplified flow cytometry plots. The measurements were performed once. Endogenous miR-21 in HCT-116 cells was used to measure the On state, and LNA-21 inhibitor was used to measure the Off state.

© 2016 The Authors
